# Development, Pre-Testing and Feasibility Testing of Multi-Component Interventions, Critical for Mental Health Promotion in Primary Care among Mexican-American Adolescents Living in Rural America

**DOI:** 10.3390/children10030465

**Published:** 2023-02-26

**Authors:** Jane Dimmitt Champion

**Affiliations:** School of Nursing, The University of Texas at Austin, Austin, TX 78701, USA; jdchampion@mail.nur.utexas.edu

**Keywords:** adolescent, rural, primary care, behavioral interventions

## Abstract

Rural America is often viewed as bereft of social problems facing urban America. Rural families, however, experience stressors due to low employment rates, fewer educational opportunities, a relatively increased incidence of poverty and limited access to mental health care. These families are at increased risk for substance use, violence and associated psychological distress that occurs when failing to cope with stress. Rural children, experiencing these stressors and affected by barriers of culture, poverty and remote access to mental health care, are at higher risk for these negative health outcomes. The need for culturally appropriate intervention tailored to the target population, rural Mexican-American adolescent women, is consistently supported by evidence. A one-size-fits-all approach most likely will not effectively impact behavior and health outcomes. The fact that few studies evaluated effects of mental health interventions on multiple outcomes including substance use, violence, unintended pregnancy and STI is problematic given previously noted associations. Evidence is needed to evaluate associations between mental health interventions and these outcomes. This manuscript presents results of preliminary studies, conducted using a multi-method research approach for development, pre-testing and feasibility testing of interventions for rural primary care settings. This methodology is appropriate when dealing with the complexity of social phenomena. It provides a look at an issue from all angles and thereby the cultural context and perspective informing intervention development. These multi-component interventions are critical for mental health promotion among Mexican-American adolescent women living in rural America.

## 1. Introduction

Forty-six million Americans, or 15 percent of the population, live in rural areas and often have difficulty obtaining needed health care services. The U.S. Census Bureau defines rural as what is not urban—that is, after defining individual urban areas, rural is what is left [1]. Rural America is often viewed as bereft of the social problems facing urban America. Rural families, however, experience stressors due to low employment rates, fewer educational opportunities, a relatively higher incidence of poverty and limited access to mental health care [2,3,4]. These families are at increased risk for substance use, violence and associated psychological distress that occurs when failing to cope with stress. Rural children, experiencing these stressors and affected by barriers of culture, poverty and remote access to mental health care, are at higher risk for these adverse health outcomes [5,6,7].

HIV is increasing in rural, minority and adolescent populations [8]. Relationships between substance use and behaviors that result in unintended pregnancy, violence and sexually transmitted infection (STI) among adolescent women have been described [9,10]. Studies indicate that adolescents may be particularly vulnerable to these adverse outcomes because of a lack of experience on which to make decisions or a lack of family or community support [11,12]. Considerable evidence documents that substance use, violence, and behaviors resulting in unintended pregnancy or STI is associated with increased medical utilization [13]. Increased medical utilization may be seen as protective in promoting adolescent health-seeking behavior; however, potential mediating factors such as family or social support, community environment and availability of mental health access impacts utilization of these resources in rural America [14,15].

Previous research with rural adolescents attempted to characterize differences that may exist as a result of community environments [16,17]. Rural Mexican-American adolescents (RMAA) are disproportionately at risk for contracting STI without adequate knowledge [18,19,20,21]. The birth rate among RMAA continues to be among the highest in the nation [10,22,23]. This relatively higher birth rate reflects the large population of youth exposed to potential STI transmission [24,25]. Proportionately more RMAA live in the US-Mexico border regions in which the STI diagnoses are among the highest in the US. The highest substance use, adolescent pregnancy and STI rates are also found in the US-Mexico border regions among Mexican-American populations [10,22,23]. These factors place Mexican-American adolescent women living in rural communities at high risk for these health disparities and without access to mental health resources in rural health facilities.

## 2. Background

Findings indicate that rural adolescents have higher odds of risk behaviors including sexual intercourse and a history of unintended pregnancy in comparison to their urban or suburban counterparts [11]. Many RMAA have sexual or physical violence, substance use and pregnancy histories [26,27]. RMAA parents are potentially at higher risk of violent behavior [23]. Unfortunately, confidentiality concerns inhibit reporting of sexual or physical abuse occurring in rural communities [2,3]. These findings are important in identifying unique characteristics and needs of rural populations that service providers can address in primary care-based mental health interventions. Findings reinforce the need to focus on relationships between family and community environment and adolescent risk and protective behaviors for health promotion [28,29,30]. Determining unique issues for mental health interventions among RMAA becomes particularly problematic because little research exists. To create effective interventions for RMAA, interventions must be sensitive to the complex realities of their lives and offer realistic alternatives that will allow them to protect themselves. Despite growing literature documenting that health promotion interventions have proven successful in well-controlled research settings, few interventions have been effectively implemented in primary care-based settings and in locations serving ethnic minority, low-income, rural populations facing health disparities. RMAA represent a vulnerable population at risk for health disparities including substance use, violence, unintended pregnancy and STI. The presence of these risks among an increasing Mexican-American population living in the United States with limited and remote mental health care access identifies a significant need for health promotion for prevention of adverse health outcomes. Inclusion of multi-component mental health promotion interventions within primary care-based settings promotes acceptability and accessibility of these interventions within rural communities particularly among those concerned with issues of confidentiality. The testing and dissemination of multi-component interventions within rural primary care-based clinics for utilization among a population with enormous health care needs and a paucity of resources creates a significant impact in a time of great need [20,29,31,32]. The Rural Health Clinic Services Act of 1977 [33] was enacted to address an inadequate supply of physicians in rural areas and to increase the utilization of non-physician practitioners such as nurse practitioners in rural areas. There are approximately 4500 rural health clinics nationwide providing access to primary care services in which these mental health promotion interventions may be disseminated.

## 3. Materials and Methods

The following section includes details regarding the multi-method research approach used for development, pre-testing and feasibility testing of multi-component interventions for mental health promotion in primary care among Mexican-American adolescents living in rural America. These descriptions detail the processes for development of the original evidence-based interventions, Project Safe and Project Image, subsequently modified for primary care interventions with RMAA.

## 4. Project Safe and Project Image: Evidence-Based Interventions

Extensive ethnographic fieldwork was conducted to develop culturally and linguistically appropriate cognitive behavioral interventions for low-income minority women [9,14,34,35,36,37]. Qualitative data focused on this target population’s risk perceptions, values and beliefs, knowledge and concerns about STI, behaviors and communication, partner relationships and strategies to motivate commitment to behavior change. Results provided preliminary insight into how to encourage health promotion through risk recognition, motivating change and identifying barriers. Findings were integrated to create gender- and culture-specific interventions for urban ethnic minority (African- and Mexican-American) women (Project Safe) [38] and subsequently ethnic minority (African- and Mexican-American) adolescent women (Project Image) [9,14,39]. These cognitive behavioral interventions promoted health by reducing risk behavior via counsel from a multi-ethnic team. The interventions (Project Safe and Project Image) were found to be effective and designated as such by the DHHS (Office of Adolescent Health) and CDC (Evidence-based Behavioral Interventions) [39].

Theoretical Framework. The Health Promotion Model (HPM) was adapted to guide qualitative data collection, intervention construction and overall research design and questionnaire development for outcomes measurement. The model builds on and integrates elements of several social psychological theories, refs. [40,41] such as the Health Belief Model, self-efficacy theory, decision-making models and diffusion theory.

Health Promotion Model: Rural Mexican-American Adolescent Mental Health Promotion. Previous work informed an initial understanding of the configuration of psychosocial and situational factors that are associated with primary outcome variables including substance use, violence, unintended pregnancy and STI among RMAA for intervention development. Antecedent explanatory factors include psychosocial and situational factors that may work through HPM constructs to affect intermediate and subsequently primary outcomes addressed in the RMAA intervention.

Translation of Intervention for RMAA. Ethnographic techniques used for development and testing of Project Safe and Project Image were applied in the multi-method preliminary studies to guide RMAA intervention development and testing to further adapt the HPM to focus on substance use, violence, unintended pregnancy and STI. The multi-method research approach includes multiple quantitative and qualitative methodologies. This research approach is appropriate when dealing with the complexity of social phenomena. It provides a look at an issue from all angles and thereby the cultural context and perspective to inform intervention development. The following preliminary work identified psychosocial and situational factors that impact behavior and subsequently impact substance use, violence, unintended pregnancy and STI. These multi-method studies combined qualitative and quantitative methodologies for intervention translation for RMAA. Complex phenomena such as sexual behavior is not one-dimensional, particularly in a group of potential participants that are vulnerable to a wide variety of influences. These studies utilized multiple data gathering techniques; semi- and structured interviews and substance use, violence, pregnancy and STI screening for implementation and evaluation of intervention effectiveness. The HPM theoretical framework and associated measurement was applied successfully throughout these studies thereby strengthening this approach and providing an opportunity to make valid comparisons of behavioral and biological outcomes. Feasibility testing of this process in preliminary studies involved collaboration with rural primary care-based providers, families and community advocates. The following preliminary work was conducted in a five-county rural area on the Southwestern United States-Texas-Mexico border for family and community assessment for RMAA intervention modification. This work found RMAA have unique needs concerning limited mental health care access in the context of high levels of substance use, violence, unintended pregnancy and STI. These issues necessitated the implementation of unique approaches for intervention delivery. The study received Institutional Review Board approval from the University of Texas at Austin and Texas Tech University Health Science Center (IRB number: L10-106).

## 5. Results

### Preliminary STUDY

Self-concept, Violence Acculturation and Assimilation among Rural MA Adolescents. Self-concept, violence, acculturation and assimilation were assessed among rural MA adolescent men and women (*N* = 342) (aged 14–19 years) attending either alternative or traditional school settings. This unique approach provided multiple perspectives for intervention development. Overall, lower perceptions of competence including lower self-worth, academic competence, sociability and job performance were reported when compared to findings reported by urban ethnic minority adolescents. Histories of violence, frequently reported (41%), included predominately sexual (42%) violence. Adolescents reporting violence had lower perceptions of competence than those without this history. Adolescents reporting higher acculturation as measured via language use described more violence and lower perceptions of competence than those reporting lower acculturation. A need for further assessment of the essence of values, family traditions, sex roles, sources of cultural conflict and attitudes and beliefs about important areas of life including personal ambitions, religion, and fatalism or sense of lack of control was apparent. These study findings also noted the need for inclusion of content concerning violence and acculturation as a component within cognitive-behavioral interventions for RMAA [27].

Rural MA Adolescents Life Histories. Life history methods were utilized to examine the context of intimate relationships from childhood to courtship among rural MA adolescents (*N =* 342) (aged 13–19 years). This unique approach provided perspectives from adolescent men and women for intervention development. Emphasis was on interactions that were extensions of cultural expectations for intimacy, family traditions, sex roles and values. Within these life histories, trans-generational patterns of violence were seen in the constitutive patterns of learning, loving and belonging through past and present relationships. The decisions that adolescents and their families made about involvement in intimate relationships appeared to be affected by low educational levels, unemployment and poverty in this rural area. Psychological distress related to seasonal and migrant work within this impoverished area placed individuals in relationships at higher risk for substance use and violence. This information was useful for modification of cognitive behavioral interventions for interruption of cycles of violence and substance use and included a focus on the attitudes and expectations of individuals and their families. Intervention geared toward re-learning about loving within intimate relationships without these elements appeared essential. To be acceptable, these interventions must consider the concepts of acculturation and assimilation, and allow continuation of a sense of belonging within families and communities. Findings indicated that interventions positioning adolescents as outsiders would probably not be embraced. Although adolescents expressed understanding of the adverse outcomes of violent relationships and substance use, actual behavioral changes were not made. Their friends and relatives were often in relationships that were similar in context and created identification, loving and a sense of belonging. An understanding of these factors through an analysis of attitudes and expectations prior to intervention was incorporated into interventions to promote beneficial effects. Directions for further community assessment involved identification of behaviors of rural MA adolescents and their relationship to substance use, violence, unintended pregnancy and STI for further cognitive behavioral intervention development [19].

Assessment of Risk/Protective Behaviors of Rural MA Adolescents. Risk and protective behaviors of rural MA adolescents (*N =* 106) (aged 13-19 years) accessing primary care-based services were assessed. This approach provided perspectives from adolescent men and women for intervention development. All spoke English and the majority (87%) were bilingual (English and Spanish). Low levels of household income and education were described. Risk behaviors examined included four domains: substance use, psychological distress, sexual behavior and contraceptive use. Assessment of protective behaviors included the nature of parental relationships, as well as health care access and associated health care behaviors. Many reported not going to the “doctor” because “symptoms usually go away,” perhaps due to a lack of knowledge about symptoms or limited health care access. Many of the participants had misconceptions concerning pregnancy, contraception or STI. High levels of adolescent risk behavior were found (89% sexually active, 74% <15 years age at first sex, 3.65 mean lifetime partners). Less than half of the adolescents ever had STI testing; three-fourths of these believed they were not at risk. A total of 78% of sexually active women had been pregnant, and 40% more than once indicated limited use of contraception and associated unintended pregnancy. High levels of substance use (67%), and psychological distress (69%) were identified. Violence (psychological 63%, physical or sexual 43%) beginning in adolescence was frequently ongoing. Sexual partners were most frequently cited as perpetrators, thereby identifying a need for this intervention regarding interpersonal violence for RMAA. Comparisons found higher levels of psychological distress including depression, stressful situations including arguing at home, deaths, illness or injury of self or family members, insufficient emotional support, higher substance use, earlier first coitus, unintended pregnancy with difficulty deciding about an abortion and perceptions of higher risk for STI among adolescents with than without a history of violence. Adolescents experiencing violence did not report more partners, just more violence in these relationships. They were also more likely to cite humiliation or shame as a health care barrier, reflecting concerns about confidentiality in the community. Adolescents described low utilization of health care services, perhaps because of these concerns.

These adolescents’ many risk behaviors, yet few protective behaviors identified health disparities within the community. Health care barriers included access and confidentiality. Findings of risk behaviors, violence and misperceptions of risk among RMAA pointed to the need for development of multi-modal cognitive behavioral interventions that include individual counseling for mental health promotion. Conclusions were that RMAA would benefit from individual counseling to provide confidential assessment for appropriate and accessible intervention [15,18].

Electronic Media/Short Message Services use for RMAA Interventions. Electronic media/short message services (SMS) are now being utilized for behavioral interventions among adolescents. However, limited evidence is available regarding SMS as health promotion interventions among RMAA. To determine the utility of including SMS in these cognitive behavioral interventions, three focus groups were conducted with rural MA adolescents, ages 13–18 years who accessed rural health clinic services. Qualitative analysis identified themes. SMS use was described as primarily for communication with family and friends. SMS use for conversations about health promotion was not supported. Participants expressed a preference for face-to-face intervention. SMS did not appear to offer significant opportunity for delivery of health promotion interventions. SMS was also limited by access to these resources as costs of services resulted in short-term use. Modification of cognitive behavioral interventions for RMAA therefore included only the limited use of SMS for recruitment and retention of participants [42].

Pre-Testing of Intervention Translation. These preliminary study findings provided the foundation for translation of the intervention for RMAA. Pre-testing was offered to RMAA (*N =* 80) (aged 13–19 years) who accessed health care through a rural health clinic in the designated five-county rural area. Participants attended focus group discussions following the pre-testing, to identify necessary changes in the intervention content. Results indicated a need for a heightened focus on cultural norms concerning sexual behavior. Many had parents who never married and were teen parents. “Ex-sex” (sex with an ex-partner) and “revenge” (multiple sex partners to get even with an unfaithful partner) were common. Many were having sex when “high” or “out of control” and had sex partners who used substances and were violent. Those with previous STI had many questions regarding symptoms and risk of re-infection. The need for an emphasis on consistent contraceptive use was identified; many did not perceive a need for its use because of occasional sex. Participants did not want pregnancy yet few used contraception and if so, usually after the birth of a baby. Partners were reportedly against contraception because “… wants a baby.” Most contraception information came from friends. A need was also identified for evaluation of the psychological status of participants. Often participants received juvenile probation, experienced Child Protective Services or witnessed parental substance or partner abuse. Some previously received counseling, currently used substances or had parents with mental illness. The findings identified the following needs: (1) systematic process for assessment of mental health among RMAA through a multi-modal intervention (individual counseling, support groups, workshops) with relevant referrals to provide confidentiality and support, (2) multi-component cognitive behavioral interventions addressing contextual complexity of RMAA relationships, and (3) access via conduct of interventions within rural primary care-based clinic settings.

Feasibility Testing of Intervention Translation. The multi-component (workshop, support group, individual counseling to address substance use, violence, unintended pregnancy and STI) intervention was developed based on these findings. Feasibility testing of the intervention was conducted with RMAA (*N =* 82) (aged 13–19 years), accessing a rural primary-care based clinic in the five-county rural area. A 6-month follow-up was completed. Clinic staff provided eligible potential participants and their parents/guardians with information about the study during routine clinic visits. Study personnel contacted those who expressed interest, provided them additional information and invited them to participate; no one refused participation. Following appropriate consent or assent, participants were enrolled in the study and received the study intervention (two individual counseling, three support group and two workshop sessions). The sessions were provided by two rural health clinic nurse practitioners. Participants received >1 (100%), >2 (90%) and >4 (85%) study intervention sessions. The 6-month follow-up for intervention evaluation was high (85%). Findings supported feasibility for conduct of multi-component cognitive behavioral interventions within rural primary care-based clinic settings.

## 6. Discussion

The aforementioned multi-method studies assessed the effectiveness of translation of a unique, evidence-based, cognitive-behavioral intervention for RMAA within a rural primary care-based setting. This intervention is innovative in that it addresses multiple health disparities of high prevalence among RMAA. Rural health professionals must think, plan and act in non-traditional, innovative ways to improve the health status of rural ethnic minority communities to address these health disparities. To work with and provide services to these populations, health professionals must acquire and use knowledge of the health-related beliefs, attitudes, practices and communication patterns of RMAA and their families to improve services, strengthen programs, increase community participation and close the gap in health status among diverse population groups. A lack of accessibility and issues of confidentiality were identified as barriers to use of rural mental health services addressing substance use, violence, unintended pregnancy and STI. Rural dwellers have been described as reluctant to use mental health services for fear that they will be spotted there. They are concerned about becoming the subject of gossip [22]. These studies are innovative in that to address the particular issues related to rural isolation and confidentiality rural primary care-based clinics are advocated. These clinics are generally accessible and the services are socially acceptable. These clinics become “empowerment zones” for RMAA by incorporating mental health promotion into the routine care offered in these settings.

The multi-component interventions (substance use, violence, unintended pregnancy and STI) focus on factors influencing the development of multiple risks and on intervention that is likely to reduce the numbers of RMAAs with these problems. One of the most consistent findings in studies of adolescent behavior is that health risks are interrelated (i.e., substance use, violence, unintended pregnancy and STI) [31,43,44]. The relationships are not inevitable, but are large enough and consistent enough to justify a focus. These risk and protective factors were identified in this multi-method preliminary work for the development and feasibility testing of this mental health promotion intervention. If health promotion interventions focus on only one factor, they are not likely to have a large impact. If these interventions are to markedly reduce risk, they need to address risk and protective factors in multiple domains. This has important implications for mental health interventions. Given that multiple factors affect adolescent behavior, interventions that focus on these should be the most effective and thus are included in this cognitive behavioral intervention [1,26,45].

Feasibility testing of multi-component interventions provided in a thriving rural primary care-based clinic found that inclusion of multiple components within the intervention enhanced its acceptability for the rural primary care-based clinic setting; participation was quite robust considering there were no refusals for participation, as was the 6-month follow-up rate of 85%. Inclusion of psychoeducational workshops, support groups and individual counseling provided the flexibility to accommodate individual as well as group level interests and needs in the rural primary care-based settings. Lastly, implementation of this intervention in collaboration with rural health professionals (physicians, nurse practitioners), [13,46,47] in the rural primary care-based clinics provided a real-life setting to enhance future intervention dissemination. The feasibility of this process, successfully tested in multi-method preliminary studies provides a solid foundation to ensure its successful implementation.

## 7. Conclusions

The need for cognitive-behavioral interventions tailored to the target population is consistently supported by evidence. A one-size-fits-all approach most likely will not effectively impact behavior and health outcomes. The fact that few studies evaluated the effect of cognitive-behavioral interventions on multiple outcomes including substance use, violence, unintended pregnancy and STI is problematic given previously noted associations. Evidence is needed to further evaluate associations between multi-component cognitive-behavioral interventions and these outcomes in rural America. Continued identification of explanatory variables, modifiable intermediate outcomes and testing of associated multi-component interventions within primary care settings, as initiated in this study, is critical for mental health promotion among RMAA.

## Data Availability

The data presented in this study are available on request from the corresponding author. The data are not publicly available to protect the privacy of research participants by prohibiting disclosure of identifiable, sensitive research information to anyone not connected to the research except when the participant.
